# Cultivation of *Anabaena* sp. at Different Phosphorus Excess Concentrations: Growth Parameters, Value‐Added Metabolites, and Microcystin‐LR Production

**DOI:** 10.1111/ppl.70632

**Published:** 2025-11-10

**Authors:** Fatemeh Rostami, Omidvar Farhadian, Nasrollah Mahboobi Soofiani, Mahmood Etebari, Amir Mahboubi

**Affiliations:** ^1^ Department of Natural Resources Isfahan University of Technology Isfahan Iran; ^2^ Department of Pharmacology and Toxicology Isfahan University of Medical Sciences Isfahan Iran; ^3^ Swedish Centre for Resource Recovery University of Borås Borås Sweden

**Keywords:** antioxidant, cyanotoxins, *Daphnia magna*, fatty acids, phosphorus

## Abstract

Phosphorus is a key driver of cyanobacterial proliferation and the production of secondary metabolites in freshwater ecosystems. In this study, the effects of different phosphorus concentrations, including 7.10 (control treatment), 7.74, 8.38, and 9.66 mg L^−1^ (CP_230_, TP_250_, TP_271_, and TP_312_, respectively), on growth, valuable biochemicals, as well as the toxicity potential of *Anabaena* sp. in BG‐11 medium were investigated. Elevated phosphorus levels significantly enhanced chlorophyll *a* and total carotenoid contents, with the highest values recorded under 8.38 mg P L^−1^ (1.16 ± 0.04 mg L^−1^ and 1.07 ± 0.30 mg L^−1^, respectively). This treatment also yielded the greatest total phenolic content (8.04 ± 0.32 mg GAE g^−1^ DW^−1^) and antioxidant activity (IC_50_ = 2.98 ± 0.02 mg mL^−1^). Antibacterial assays demonstrated notable inhibition zones against *Escherichia coli* (9.30 ± 1.20 mm) and *Staphylococcus aureus* (4.50 ± 0.90 mm) in the treatment with 8.38 mg P L^−1^. GC/MS analysis showed that the cyanobacterial extracts contained several biomolecules such as phenol, 2,4‐Di‐tert‐butylphenol, hexadecanoic acid methyl ester, and other compounds with antioxidant and antibacterial activity. Palmitic acid, palmitoleic acid, oleic acid, and linoleic acid were the dominant fatty acids in the lipid profile of *Anabaena* sp. In addition to its antibacterial properties, *Anabaena* sp. showed moderate to low toxicity against *Daphnia magna* , depending on the phosphorus concentration of the treatments. The findings of the current research indicated that *Anabaena* sp. not only produces cyanotoxins but also beneficial chemicals, positioning it as a potential new target for antibacterial and antioxidant drug development.

## 1 Introduction

Aquaculture is one of the most important sectors for food production globally; however, challenges like disease attacks and water pollution hinder its growth. Utilizing algae is a promising alternative to deal with these environmental problems and enhance aquatic production (Vijayaram et al. 2024). Algae and cyanobacteria are vital in aquaculture due to their roles as aquatic feed as well as their potential in treating the wastewater of cultivation systems. While they provide essential nutrients and contribute to the food web, certain species can produce harmful toxins or negatively impact the taste of fish (Aklakur et al. 2023). In this regard, cyanobacteria produce metabolites that can be classified as either primary or secondary. Primary metabolites are essential for survival, whereas secondary metabolites are biosynthesized in response to biotic and abiotic stimuli (Kultschar and Llewellyn 2018). Secondary metabolites serve auxiliary functions such as defense mechanisms against other organisms, stress response, signaling, the transport of metals, photoprotection, antioxidant activity, and facilitators of symbiosis (Kultschar and Llewellyn 2018; Jones et al. 2021).

Cyanobacterial secondary metabolites exhibit a range of properties, being classified as either toxic or non‐toxic. Non‐toxic metabolites produced by cyanobacteria are acknowledged as natural bioactive substances with a wide range of pharmaceuticals and industrial applications (Demay et al. 2019). Also, these microorganisms are recognized for their ability to generate a diverse array of toxic secondary metabolites (Huang and Zimba 2019). Microcystins, anatoxin‐A, saxitoxin, and yessotoxin are a few cytotoxins produced by cyanobacteria (Haque et al. 2017). Among them, microcystins, particularly hepatotoxins, are extensively studied due to their global impact. Overall, cyanobacterial metabolites are categorized into 14 classes according to their biological activity. These activities encompass hepatotoxicity, antioxidant, antiviral, dermatotoxicity, lethality, neurotoxic activity, cytotoxicity, anti‐inflammatory responses, antibacterial, antialgal properties, antifungal, antiprotozoal activity, and inhibitory effects on enzymes (Demay et al. 2019; Polyak and Sukharevich 2023). Therefore, cyanobacteria function as natural sources of both beneficial and harmful metabolites.

Researchers have demonstrated that abiotic factors including nutrient concentration, light, temperature, carbon sources, salinity level, and pH affect metabolite synthesis performance in cyanobacteria. For instance, nutrient stress induces the production of free radical species within the cell, potentially altering the content of antioxidants (Goiris et al. 2015). Recent studies have investigated the impact of macronutrients, including nitrate, phosphate, and sulfate, on the growth of cyanobacteria and their biochemical constituents, such as protein, lipid, phycocyanin, polyhydroxyalkanoates, and cyanophycin (Canizales et al. 2023; Anh Nguyen et al. 2024; Chung and Ng 2024; Kharwar and Mishra 2024; Saini and Mona 2024). Hence, regulating environmental factors and nutrient media components is essential for growth optimization as well as production and accumulation of metabolites in cyanobacteria (Yalcin 2020; Assunçao et al. 2023). This involves careful adjustment of variables such as nutrient concentration, illumination, and implementation of stress conditions (Odenthal et al. 2024). Phosphorus is essential for algae growth, fat production, fatty acid production, and metabolic processes, including energy transfer, message transfer, and photosynthesis (Ota et al. 2016; Yang et al. 2018). In general, to maintain algal growth, approximately 0.03%–0.06% phosphorus in the culture medium is required, which makes up a little less than 1% of the total algal biomass (Procházková et al. 2014; Ota et al. 2016). Previous investigations into cyanobacteria have largely concentrated on phosphorus deficiency and its physiological consequences. For instance, Tripathi et al. (2013) demonstrated that under inorganic phosphate limitation in *Anabaena fertilissima*, the cellular phosphorus quota declined rapidly, while both cell‐bound and cell‐free alkaline phosphatase activities were strongly induced to scavenge alternative P sources (Tripathi et al. 2013). Similarly, Jonna et al. (2015) employed transcriptomic and proteomic profiling of *Anabaena* sp. under phosphorus deprivation, observing a significant upregulation of phosphate transporters and phosphate assimilation genes, which indicates extensive molecular adaptation to low‐P stress (Jonna et al. 2015). However, despite this solid foundation regarding low‐P responses, the metabolic and regulatory impacts of excessive or supra‐optimal phosphorus concentrations remain poorly understood. This study aims to investigate the effect of phosphorus‐enriched media on the growth parameters and metabolites of *Anabaena* sp., a nitrogen‐fixing cyanobacterium (Videau and Cozy 2019). *Anabaena*'s metabolites possess various medicinal properties, including antimicrobial activity (Chauhan et al. 2010), antioxidant, antitumor, and larvicidal properties (Chauhan et al. 2010; Suhail et al. 2011). This species also plays a role in bioremediation and biofertilizer production and can be used in bioenergy production (Stebegg et al. 2012). Additionally, there is an acknowledged knowledge gap in cyanobacterial research, where laboratory‐scale studies often focus on the impact of nutrients either on the beneficial metabolites produced by cyanobacteria or on their toxins and harmful blooms. The current work considers the effects of different phosphorus concentrations on both beneficial bioactive compounds and toxin molecules of *Anabaena* sp., concurrently. This research intends to report (i) biomass production, (ii) potentially beneficial metabolites (e.g., lipids, polyphenols, antibacterial, and antioxidant compounds), and (iii) toxic compounds (e.g., microcystins) of *Anabaena* sp. cultivated at control medium (with 230 μM P concentration) and phosphorus‐enriched media (with 250, 271, and 312 μM concentrations). Exploring the impacts of phosphorus‐enriched media on both the harmful and useful compounds of *Anabaena* sp. can expand the industrial application of cyanobacteria.

## 2 Materials and Methods

### 2.1 Culture Media

Prior to water sampling, BG‐11 liquid and agar‐solidified media were prepared. The liquid medium was formulated according to the BG‐11 recipe (Stanier et al. 1971). For the preparation of the agar‐solidified petri dish medium, 7.5 g of agar was added into 500 mL of BG‐11 liquid medium and autoclaved at 121°C for 20 min. The autoclaved medium was decanted into petri dishes and allowed to cool at room temperature. Both liquid and solid media were preserved in a refrigerator at 4°C until the commencement of the experiments. The nutrient components of BG‐11 medium (Merck) and their concentrations are detailed in Table 1.

**TABLE 1:** BG‐11 medium ingredients (Stanier et al. 1971).

		Stocks	Concentration (g L^−1^)
Macroelement nutrients	1	NaNO_3_	150.00
	2	K_2_HPO_4_	4.00
	3	MgSO_4_.7H_2_O	7.50
	4	CaCl_2_. 2H_2_O	3.60
	5	Citric acid	0.60
	6	Ammonium ferric citrate green	0.60
	7	EDTANa_2_	0.10
	8	Na_2_CO_3_	2.00
Microelement nutrients	9	H_3_BO_3_	2.86
		MnCl_2_.4H_2_O	1.81
		ZnSO_4_.7H_2_O	0.22
		Na_2_MoO_4_.2H_2_O	0.39
		CuSO_4_.5H_2_O	0.08
		Co(NO_3_)_2_.6H_2_O	0.05
		Medium	Per liter
		Stock solutions 1–8	10 mL of each
		Stock solution 9	1.0 mL

### 2.2 Cyanobacterium Isolation

Water samples were collected from the water surface of the Zayandeh Roud river basin (Isfahan), a known source of cyanobacteria such as *Anabaena* sp., and subsequently transported to the fisheries laboratory at Isfahan University of Technology, Isfahan, Iran, to identify a cyanobacterial species capable of producing cyanotoxins.

To isolate the cyanobacterium, water samples were initially pipetted onto the surface of the agar medium. The inoculated agar plates were at room temperature under a 12 h light/12 h dark regime. After 10 days, the emerging colonies were transferred to sterile agar media using inoculating loops. The procedure was conducted several times to augment the population of the targeted cyanobacterium. Finally, the purified colonies were transferred to the BG‐11 liquid medium and incubated under continuous illumination of white fluorescents (37 μmol m^−2^ s^−1^), constant aeration using an air stone connected to an aquarium pump (100 mL min^−1^), and at a temperature of 25°C ± 2°C until reaching stationary phase (Shawer et al. 2023). The isolated cyanobacterium was identified as *Anabaena* sp. species according to its morphological features.

### 2.3 Experimental Design and Implementation

The experiments were conducted with four experimental groups differentiated by phosphorus concentration. For this purpose, BG‐11 liquid medium with 7.10 mg P L^−1^ was considered as the control group. Phosphorus concentrations were adjusted by increasing 9%, 18%, and 36% of the standard phosphorus level of BG‐11 culture medium [Approximately the basic phosphorus of the culture medium (7.1 mg *P* ^−1^) with an increase of 10%, 20%, and 40%] to assess the physiological response of *Anabaena* sp. to incremental P enrichment. Accordingly, BG‐11 liquid media with 7.74, 8.38, and 9.66 mg P L^−1^ were prepared to establish phosphorus‐enriched treatment groups. The molar concentrations for the control and treatment groups were calculated as 229.3 (≈230), 249.8 (≈250), 270.5 (≈271), and 311.9 (≈312) μM. According to their phosphorus concentrations (μM), the groups were designated as CP_230_ for the control and TP_250_, TP_271_, and TP_312_ for the treatment groups, respectively.

**FIGURE 1:**
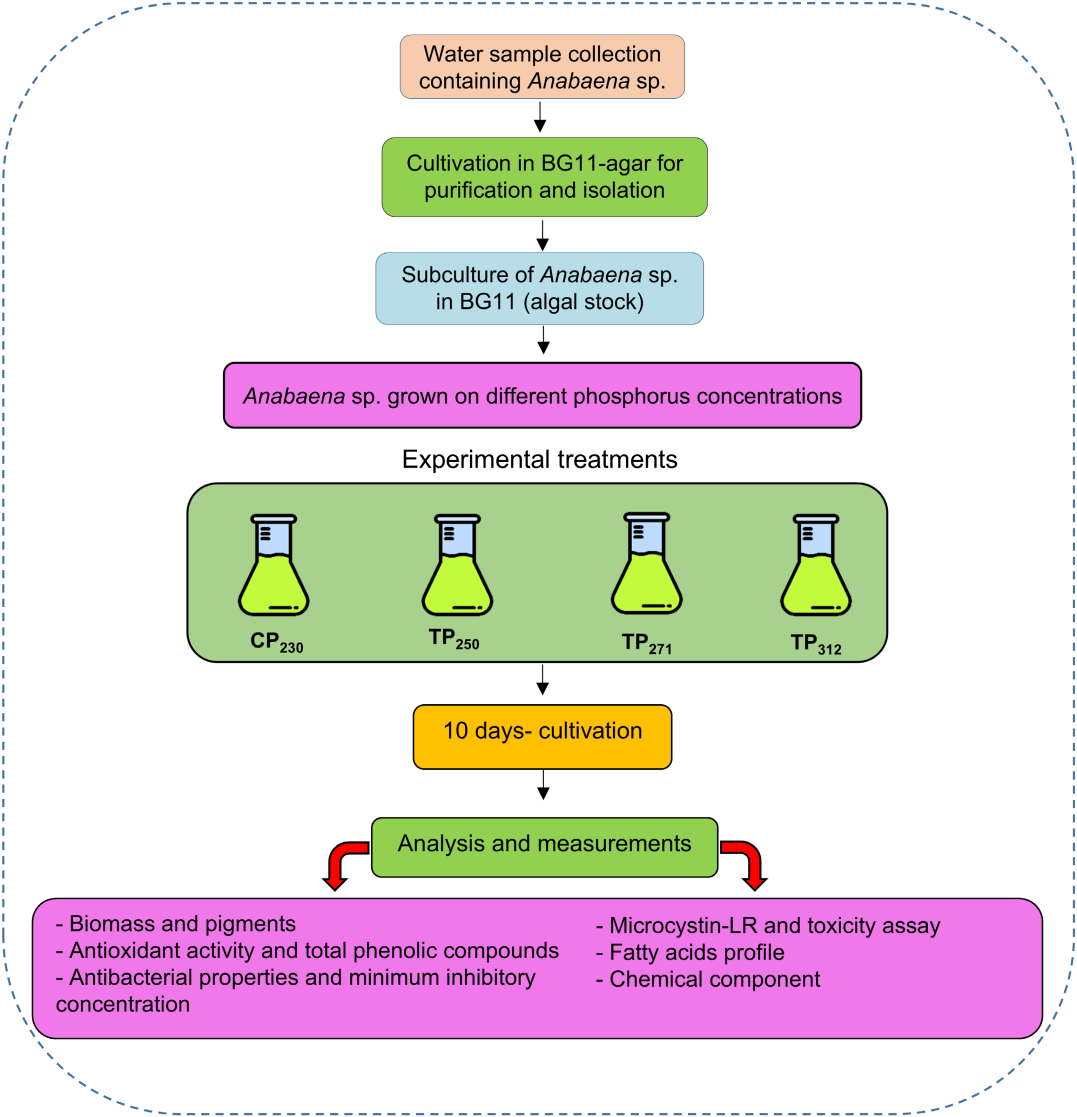
A schematic flow diagram summarizing the experimental design of the present study.

To initiate the experiment, 1400 mL of CP_230_, TP_250_, TP_271_, and TP_312_ media were added to 2000 mL Erlenmeyer flasks. Following this, 300 mL of the prepared *Anabaena* sp. stock (based on OD 0.2 after inoculation), at exponential phase, was poured into each culture container. Erlenmeyer flasks were maintained in the culture room under the identical cultural conditions specified in Section 2.2. The effect of cultivation media with different phosphorus concentrations on cyanobacterium growth, along with their beneficial metabolites and toxin production, was examined, as detailed in the following sections. A summary of the experimental design of the present study is shown in Figure 1.

### 2.4 Cyanobacterium Growth

The effect of phosphorus concentrations on cyanobacterial growth was monitored by determining dry biomass concentration (g L^−1^). After 10 days, the cultures were centrifuged at 5480 × *g* for 5 min to collect the harvested cyanobacterial biomass. The harvested wet pellets were desiccated in an oven at 60°C for 24 h. The mass of dried biomass was quantified to determine biomass production in the control and treatment groups based on mass concentration (g L^−1^) directly.

### 2.5 Extraction and Determination of Pigments

Chlorophyll *a* (Chl *a*) and total carotenoids were quantified at the end of the experiment, on day 10. For this purpose, 10 mL of the cultures were sampled and centrifuged for 15 min at 2265 × *g*. Then, the supernatant was discarded, and 10 mL of 90% methanol was added to the centrifugal tubes containing *Anabaena* sp. pellets. The resulting mixture was vortexed for 1 min. Thereafter, the tubes were placed in an oven at 60°C for 10 min. Afterward, the tubes were centrifuged for an additional 15 min at 2265 × *g* to separate biomass residual. Eventually, the absorbance of the supernatant consisting of pigments was recorded at the wavelengths of 665 and 720 nm for Chl *a* and wavelengths of 470 and 720 nm for total carotenoid using a UV‐spectrophotometer (JENWAY 6400, Bibby. Scientific Ltd.). The amounts of pigments (μg L^−1^) were determined according to the following equations (Wellburn 1994; Ritchie 2006; Zavrel et al. 2015):

(1) $$\mathrm{Chl}\;a\;\left(\mathrm{mg}\;L^{-1}\right)=12.9447\;\left(A_{665}-A_{720}\right)$$

(2) $$\text{Total carotenoids}\;\left(\mathrm{mg}\;L^{-1}\right)=\left(1000\;\left(A_{470}-A_{720}\right)\right)-2.86\;\left(\left(\mathrm{Chl}\;a\right)/221\right)$$

### 2.6 Lipid Extraction and Fatty Acid Composition

The modified Bligh and Dyer method (Bligh and Dyer 1959), as established by Nezafatian et al. (2024), was applied to extract total lipids from the dried biomass of *Anabaena* sp. Lipids from *Anabaena* sp. underwent transesterification to provide fatty acid methyl esters (FAME), which were subsequently analyzed via gas chromatography (Agilent 6890, Agilent Technologies). The gas chromatograph featured a flame ionization detector (FID) and a MODEL capillary column with dimensions of 100 m length × 0.25 mm internal diameter and a film thickness of 0.2 μm. The oven temperature was initially set at 165°C, and it was elevated to 210°C at a rate of 5°C min^−1^. Finally, the temperature increased to 280°C for 5 min at the same rate of 5°C min^−1^. The temperatures of the injector and detector were maintained at 280°C and 320°C, respectively. Helium served as the carrier gas, maintaining a flow rate of 1 mL min^−1^.

### 2.7 Crude Methanolic Extract

The crude extracts of *Anabaena* sp. biomass were prepared utilizing 99% methanol (Merck). Initially, 1 g of freeze‐dried biomass was incorporated into 50 mL of methanol. The amalgamation was agitated by an orbital rotator shaker (100 rpm) at ambient temperature (25°C ± 2°C) for 24 h. The resulting extracts were centrifuged at 2265 × *g* for 10 min. The extraction process was subsequently done four times, and all the supernatants were combined. Methanol was ultimately evaporated using a rotary evaporator (Heidolph Hei‐VAP) to concentrate the extracts. The extracts were stored in a −20°C freezer before analysis.

#### 2.7.1 Total Phenolic Content of Extracts

The total phenolic content of *Anabaena* sp. extracts was assessed utilizing the Folin and Ciocalteu method with some modifications. Briefly, 0.5 mL of each methanolic extract sample was mixed with 5 mL of Folin–Ciocalteu reagent in the test tubes, which had been previously diluted tenfold with distilled water. The resulting solution was thereafter maintained at ambient temperature for 5 min. Subsequently, 100 μL of sodium carbonate solution (75 g L^−1^) was added to the mixture. The resultant mixture was incubated for 90 min at ambient temperature in the absence of light. The absorbance of each sample was ultimately measured at 765 nm utilizing a spectrometer (JENWAY 6400). A standard calibration curve was established by serially diluting a gallic acid solution with concentrations from 0 to 100 mg L^−1^. The phenolic content was expressed as milligrams of gallic acid equivalents per gram of dry weight of biomass (mg GAE g^−1^ DW^−1^) (Barroso et al. 2016).

#### 2.7.2 Antioxidant Activity of Extracts

The antioxidant efficacy of *Anabaena* sp. extracts was evaluated using the 2,2‐diphenyl‐1‐picrylhydrazyl (DPPH) technique described by Li, Han, et al. (2014) with some modifications. 200 μL of the methanolic extracts of *Anabaena* sp. was added to 2.8 2.8 mL of 0.1 mM DPPH methanolic solution. The obtained samples were kept for 15 min in the dark at room temperature. The control sample was the DPPH solution without extract. Thereafter, the absorbance was measured using a UV‐spectrophotometer (UV‐spectrophotometer 2401PC, Shimadzu Corporation) at a wavelength of 517 nm. Finally, the percentage of free radical inhibition was calculated by measuring the amount of DPPH color change from purple to yellow using the following formula:

(3) $$\text{DPPH scavenging activity}\;\left(\%\right)=\left(A_{0}–A_{1}/A_{0}\right)\times 100$$

In which A_0_ is the absorbance of the control (DPPH and methanol) and A_1_ is the absorbance of the sample.

Different concentrations (0.01–0.08 mg L^−1^) of ascorbic acid (vitamin C) were used as standard solutions. For this purpose, concentrations of ascorbic acid and the DPPH radical scavenging percentage were calculated. These percentages were plotted on the y‐axis against the ascorbic acid concentrations on the x‐axis to construct a standard curve. Using this curve, the absorbance (or scavenging percentage) of the sample was converted to mg L^−1^ of ascorbic acid equivalents.

#### 2.7.3 *Antibacterial* Efficacy of Extracts

##### 2.7.3.1 Disc Diffusion Assay

The antibacterial activity of *Anabaena* sp. extracts, grown at different phosphorus concentrations, was evaluated using two strains of Gram‐negative *Escherichia coli* MTCC 1687 and Gram‐positive *Staphylococcus aureus* MTCC 96 bacteria by the disk diffusion method (Abd El‐Aty et al. 2014). The desired bacterial strains were obtained from the Biotechnology Research Institute at Isfahan University of Technology (Isfahan, Iran), and incubated in nutrient agar medium at 37°C for 24 h. Plates containing Mueller‐Hinton culture medium (Merck; Cat. No. 105437) were prepared and the 0.5 McFarland suspension (1 × 10^8^) prepared from the bacterial strains was swabbed on the prepared cultures. Subsequently, the discs treated with methanol extract were positioned on the bacterial culture plates and maintained at room temperature for 5 min to facilitate diffusion. Following 24 h of incubation at 37°C, the diameter of the formed halos was measured. Methanol served as the negative control, whereas gentamicin antibiotics functioned as the positive control in this study.

##### 2.7.3.2 Minimum Inhibitory Concentration Assay

The minimum inhibitory concentration (MIC) of *Anabaena* sp. cyanobacterial extracts was evaluated using two sterile 96‐well plates (each for one bacterial strain) and broth microdilution technique. In this manner, 100 μL of Mueller‐Hinton broth culture medium was poured into seven wells of the first three rows (representing triplicate) in each microplate. Then, 100 μL cyanobacterial extract at a concentration of 20 mg DW L^−1^ of *Anabaena* sp. biomass was added into the first well of each row. After proper mixing, 100 μL of the mixture was transferred into the next well. The process was repeated for the next 3 wells, resulting in 10, 5, 2.5, 1.25, and 0.625 mg mL^−1^ extract concentrations in each well in order. Then, 100 μL of the prepared bacterial stock (10^8^ CFU mL^−1^) was added to all wells of the microplate. The well number 6 in each row contained only culture medium and extract as an extract control, while wells 7 in each row contained culture medium and bacteria as a bacterial control to determine bacterial turbidity. In the final step, the microplate was placed in an incubator at 37°C for 24 h, after which the MIC was assessed by comparing the turbidity of the treated wells to that of the control wells. The lowest concentration of each test solution that prevented the development of any of the microorganisms in the wells was recorded as the MIC value (Nayeem et al. 2023).

### 2.8 Analysis of Microcystin‐LR and Toxicity Assay

The microcystin content of *Anabaena* sp. extracts was measured using an ELISA kit (Fan et al. 2022). The test was carried out in three replications and read using an ELISA reader at a wavelength of 450 nm (Fan et al. 2022). The results were expressed as pg. of MC‐LR per g of dry biomass.

The toxicity assay of *Anabaena* sp. extract was conducted using *Daphnia magna* , a freshwater zooplankton species. For this purpose, a certain dried powder of *Anabaena* sp. biomass (harvested from CP_230_, TP_250_, TP_271_, and TP_312_ cultures) was added to the culture medium of *D. magna* to prepare mixtures with concentrations of 0, 250, 500, and 1000 mg L^−1^. The mixtures were soaked for 24 h, and subsequently were subjected to sonication (20 kHz) for 15 min. After that, the suspensions were centrifuged (1790 × *g* for 15 min) to obtain the aquatic extracts. Acute toxicity was assessed by adding 10 individuals of *D. magna* per replication into 30 mL of the aqueous extract of *Anabaena* sp.

The *D. magna* culture media served as a negative control. Green algae ( *Chlorella vulgaris* ) were utilized as sustenance in all treatments. The survivors in each test tube were recorded every 4 h during the initial 24 h and then recorded daily until the fourth day of exposure. All physicochemical characteristics of water and the surroundings, including pH, temperature, and other aspects, were maintained constant throughout the experiment (Ferrao‐Filho et al. 2014). The lethality percentage of *Anabaena* sp. aqueous extract and control treatment was determined through statistical analysis of *D. magna* survival and LC50 using probit analysis software (Regueiras et al. 2018).

(4) $$\begin{array}{c} \text{Mortality}\;\left(\%\right)=(\text{Number of deceased}\;D.\;\text{magna}/\\ \text{Initial population of live}\;D.\;\text{magna})\times 100. \end{array}$$

### 2.9 Identification of Chemical Compounds

Identification of chemical compounds of *Anabaena* sp. was done using a GC–MS device (GC‐Agilent 5890B, MS‐5977A, Agilent Technologies). This device was equipped with a Teledyne Tekmar Stratum Purge and Trap Concentrator (Model 14–9800‐200), mass detector, capillary column HP‐5MS (column length 30 m, column inner diameter 250 mm, and film thickness 0.25 μm). Helium gas with a flow of 21.7 mL min^−1^ and a pressure of 14:30 psi was used as a carrier gas. Processing was done using ChemStation software. The percentage of compounds in the samples was determined by calculating the area under the GC–MS chromatogram.

### 2.10 Statistical Analysis

The experiments were carried out employing a fully randomized design with three replications. The analysis involved one‐way ANOVA, presenting the mean ± SD from three replicates. Duncan's test was employed to assess notable differences among the treatments at a 95% confidence level. The toxicity parameter (LC50) was calculated using Probit software (version 1.5). Statistical analyses were conducted using SPSS (version 26.0; SPSS Inc.) software.

## 3 Results and Discussion

Cyanobacteria produce a range of secondary metabolites that exhibit biological activities, including antimicrobial, antifungal, antiviral, and enzyme inhibitory properties, as well as toxicity to eukaryotic organisms (Carpine and Sieber 2021). This study assessed the biomass, total phenolic content, and fatty acid profile of *Anabaena* sp. cultivated under varying phosphorus concentrations. The antioxidant and antibacterial capabilities, as well as the toxic effects of *Anabaena* sp. on the zooplankton *Daphnia magna* , were also examined.

### 3.1 The Effect of Phosphorus Concentrations on Biomass and Pigment Production

#### 3.1.1 Biomass

**FIGURE 2:**
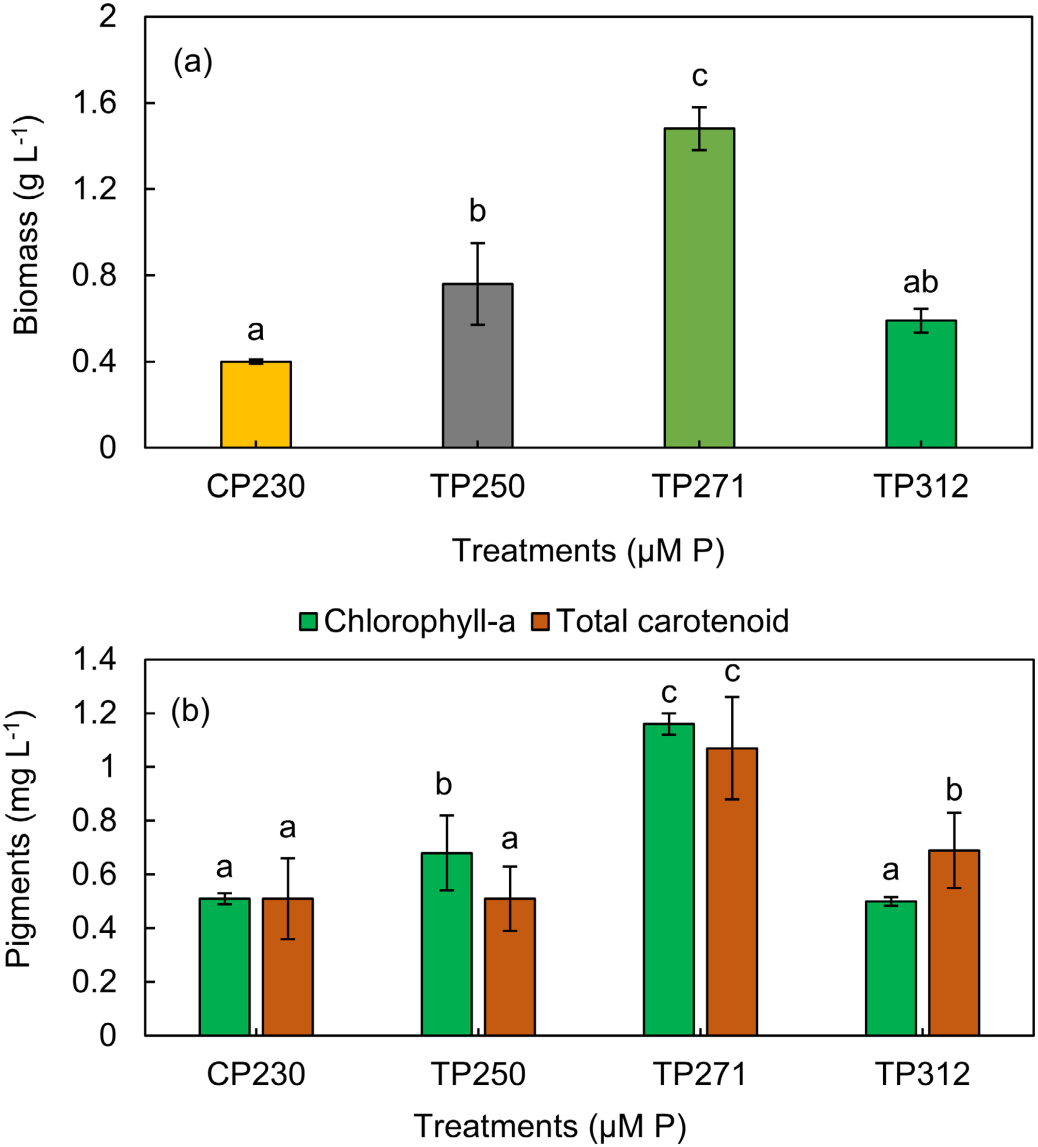
The effect of different concentrations of phosphorus on dry biomass (a) and pigments production (b) of *Anabaena* sp. Data are means ± SD from three replicates. Bars with different letters indicate significant differences according to Duncan's test (*p* < 0.05).

In the current investigation, the maximum dry biomass (1.48 ± 0.29 g L^−1^) was recorded in the TP_271_, which exhibited a significant difference from other treatments (*p* < 0.05) (Figure 2a). The dry biomasses of *Anabaena* sp. correlated with CP_230_, TP_250_, TP_271_, and TP_312_ treatments were found to be 0.40 ± 0.01, 0.76 ± 0.30, 1.48 ± 0.19, and 0.59 ± 0.05 g L^−1^, respectively. Similarly, Xin et al. (2010) reported that algal biomass concentration was approximately 30%–40% lower in low‐phosphorus cultures than in those with adequate phosphorus availability. Previous studies have shown that phosphorus stimulates the growth of microalgae; thus, providing sufficient phosphorus increases the biomass yield of microalgae (Li, Jia, et al. 2014; Fu et al. 2017). Jiao et al. (2017) stated that the lack of P led to the reduction in photosystem II (PSII) activity by influencing the electron receivers and donators in the PSII system, which significantly inhibited photosynthesis in microalgae.

Findings indicate that increasing the phosphorus concentration up to a certain level (e.g., up to 8.38 mg L^−1^ in this study) can improve algal growth and biomass concentration. Although phosphorus is an essential element for microalgal growth, excessive concentrations accumulate within cells, leading to disruption of enzymatic function and membrane permeability (Li et al. 2018). Also, it is possible that phosphorus levels above 8.38 mg L^−1^ can be growth inhibitory for *Anabaena* sp., which supports the different phosphorus requirements of various algae species as well as the direct toxicity of phosphorus or phosphate form to the species in question.

#### 3.1.2 Pigments

The pigment contents of *Anabaena* sp. cultivated under varying phosphorus levels are presented in Figure 2b. The results indicated that in *Anabaena* sp. treated with CP_230_, TP_250_, TP_271_, and TP_312_, the chlorophyll *a* concentrations were 0.51 ± 0.02, 0.68 ± 0.14, 1.16 ± 0.04, and 0.50 ± 0.01 mg L^−1^, respectively. The total carotenoid concentrations for the treatments with the same order were 0.51 ± 0.15, 0.51 ± 0.12, 1.07 ± 0.30, and 0.69 ± 0.14 mg L^−1^, respectively. Phosphorus is recognized as an essential element for the photosynthetic and physiological characteristics of microalgae (Chu et al. 2020). Phosphorus limitation results in a reduction of chlorophyll production and, therefore, a decline in the rate of photosynthesis (Rocha et al. 2018; Kumari et al. 2021). Phosphorus supports chlorophyll and carotenoid biosynthesis in cyanobacteria and microalgae by providing ATP and NADPH for key enzymatic steps such as Mg‐chelatase in chlorophyll synthesis and desaturation reactions in carotenoid formation (Fromme et al. 2010; Wu et al. 2014). Phosphorus deficiency downregulates enzymes such as phytoene synthase and chlorophyll synthase, thereby reducing pigment accumulation (Wu et al. 2014).

### 3.2 The Effect of Phosphorus Concentrations on Valuable Biocompounds and Biological Activities of Cyanobacterial Extracts

#### 3.2.1 Fatty Acids Profile

Cyanobacteria are abundant in essential fatty acids that are utilized due to their nutritional assessment values (Sharathchandra and Rajashekhar 2011). Table 2 shows the relative concentrations of monounsaturated fatty acids (MUFAs), polyunsaturated fatty acids (PUFAs), and saturated fatty acids (SFAs) in the lipid of *Anabaena* sp. biomass. In the present study, the average amount of SFAs, MUFAs, and PUFAs of the total fatty acid composition of *Anabaena* sp. was found to be 45.44% ± 4.19%, 34.79% ± 3.50%, and 17.47% ± 2.21%, respectively. In all treatments, the dominant fatty acids in the lipid profile of *Anabaena* sp. were palmitic acid (C16:0), palmitoleic acid (C16:1), oleic acid (C18:1), and linoleic acid (C18:2). Sharathchandra and Rajashekhar (2011) conducted a review of the total lipid and fatty acid composition in several freshwater cyanobacteria. C16:0 and C18:2 were identified in all examined species. Furthermore, their investigation indicated that long‐chain fatty acids including eicosenoic acid (C20:1) and lignoceric acid (C24:0) were present in lower concentrations in certain species. The experimental treatments differed significantly in terms of saturated fatty acid levels (Table 2, *p* < 0.05). The maximum concentration of MUFAs (39.01% ± 3.59%) was recorded in the TP_271_, which exhibited a statistically significant difference from other treatments (Table 2, *p* < 0.05). The concentrations of PUFAs in CP_230_, TP_250_, TP_271_, and TP_312_ treatments were 14.99% ± 1.95%, 18.20% ± 2.01%, 18.48% ± 2.25%, and 17.86% ± 2.66%, respectively. This study found that the levels of SFAs, MUFAs, and PUFAs in *Anabaena* sp. were notably affected by the concentrations of phosphorus. As the phosphorus concentration reached 8.38 mg L^−1^, there was a significant increase in MUFAs and PUFAs in comparison to the control group; however, a decline was observed at higher concentration at 9.66 mg L^−1^ compared to 8.38 mg L^−1^ P. The availability of nutrients has a considerable impact on environmental factors and the composition of fatty acids (Hu 2003; El‐Kassas 2014). Furthermore, Dahmen‐Ben Moussa et al. (2017) indicated that elevating phosphorus levels in the culture medium of the marine microalgae *Tetraselmis marina* led to a reduction in SFAs content while enhancing USFAs (MUFAs + PUFAs) content. Adequate phosphorus supply enhances the production of ATP and NADPH during photosynthesis, which are essential cofactors for the enzymatic activities of desaturases and elongases responsible for introducing double bonds and elongating fatty acid chains (Xin et al. 2010). However, excessive phosphorus beyond the optimal threshold may alter metabolic balance, potentially reducing the relative proportion of unsaturated fatty acids due to changes in carbon allocation and stress responses (Zhang et al. 2024).

**TABLE 2:** Fatty acids profile of *Anabaena* sp. in different concentrations of phosphorus.

Fatty acids composition	Treatments (μM P)			
	CP_230_	TP_250_	TP_271_	TP_312_
C14:0	1.86 ± 0.37^b^	1.80 ± 0.12^ab^	1.72 ± 0.06^a^	3.71 ± 1.02^c^
C16:0	39.00 ± 2.50^b^	38.74 ± 2.23^b^	37.55 ± 1.96^a^	37.61 ± 1.76^a^
C18:0	4.73 ± 0.35^c^	3.88 ± 0.37^b^	3.84 ± 0.51^b^	2.89 ± 0.45^a^
C20:0	0.63 ± 0.01^b^	0.49 ± 0.008^ab^	0.43 ± 0.008^a^	0.56 ± 0.019^ab^
C22:0	0.39 ± 0.006^b^	0.23 ± 0.001^a^	0.18 ± 0.011^a^	0.20 ± 0.009^a^
C24:0	0.48 ± 0.09^b^	0.45 ± 0.05^b^	0.24 ± 0.008^a^	0.18 ± 0.003^a^
C14:1	0.17 ± 0.012^a^	0.24 ± 0.05^a^	0.34 ± 0.051^b^	0.41 ± 0.09^b^
C16:1	14.35 ± 1.52^a^	16.97 ± 2.12^c^	17.65 ± 1.90^d^	15.75 ± 2.50^b^
C18: 1	12.31 ± 0.72^a^	14.03 ± 1.10^b^	16.32 ± 1.50^c^	14.59 ± 1.82^bc^
C20:1	3.15 ± 0.09^a^	3.01 ± 0.34^a^	4.09 ± 0.55^b^	4.33 ± 0.76^b^
C22:1	0.18 ± 0.001^a^	0.20 ± 0.01^a^	0.21 ± 0.006^a^	0.22 ± 0.005^a^
C24:1	ND^a^	0.10 ± 0.003^a^	0.13 ± 0.003^a^	0.14 ± 0.017^a^
C16:2	0.52 ± 0.009^b^	0.42 ± 0.005^ab^	0.43 ± 0.001^ab^	0.36 ± 0.007^a^
C18:2	13.87 ± 2.96^a^	16.98 ± 2.55^b^	17 ± 4.14^b^	16.88 ± 3.36^b^
C18:3	0.37 ± 0.002^a^	0.38 ± 0.001^a^	0.75 ± 0.009^b^	0.35 ± 0.005^a^
C20:2	0.15 ± 0.003^a^	0.23 ± 0.001^a^	0.19 ± 0.002^a^	0.17 ± 0.005^a^
C22:2	0.08 ± 0.001^a^	0.19 ± 0.005^b^	0.11 ± 0.001^a^	0.10 ± 0.002^a^
*Σ*SAFAs	47.09 ± 5.00^c^	45.59 ± 4.09^b^	43.96 ± 3.10^a^	45.15 ± 4.59^b^
*Σ*MUFAs	30.16 ± 1.75^a^	34.55 ± 4.49^b^	39.01 ± 3.59^c^	35.44 ± 4.19^b^
*Σ*PUFAs	14.99 ± 1.95^a^	18.20 ± 2.01^b^	18.48 ± 2.25^b^	17.86 ± 2.66^b^

*Note:* Data represent mean ± SE. Different letters indicate significant differences between treatments (*p* < 0.05).

Abbreviation: ND, not detected.

#### 3.2.2 Total Phenolic Content

**FIGURE 3:**
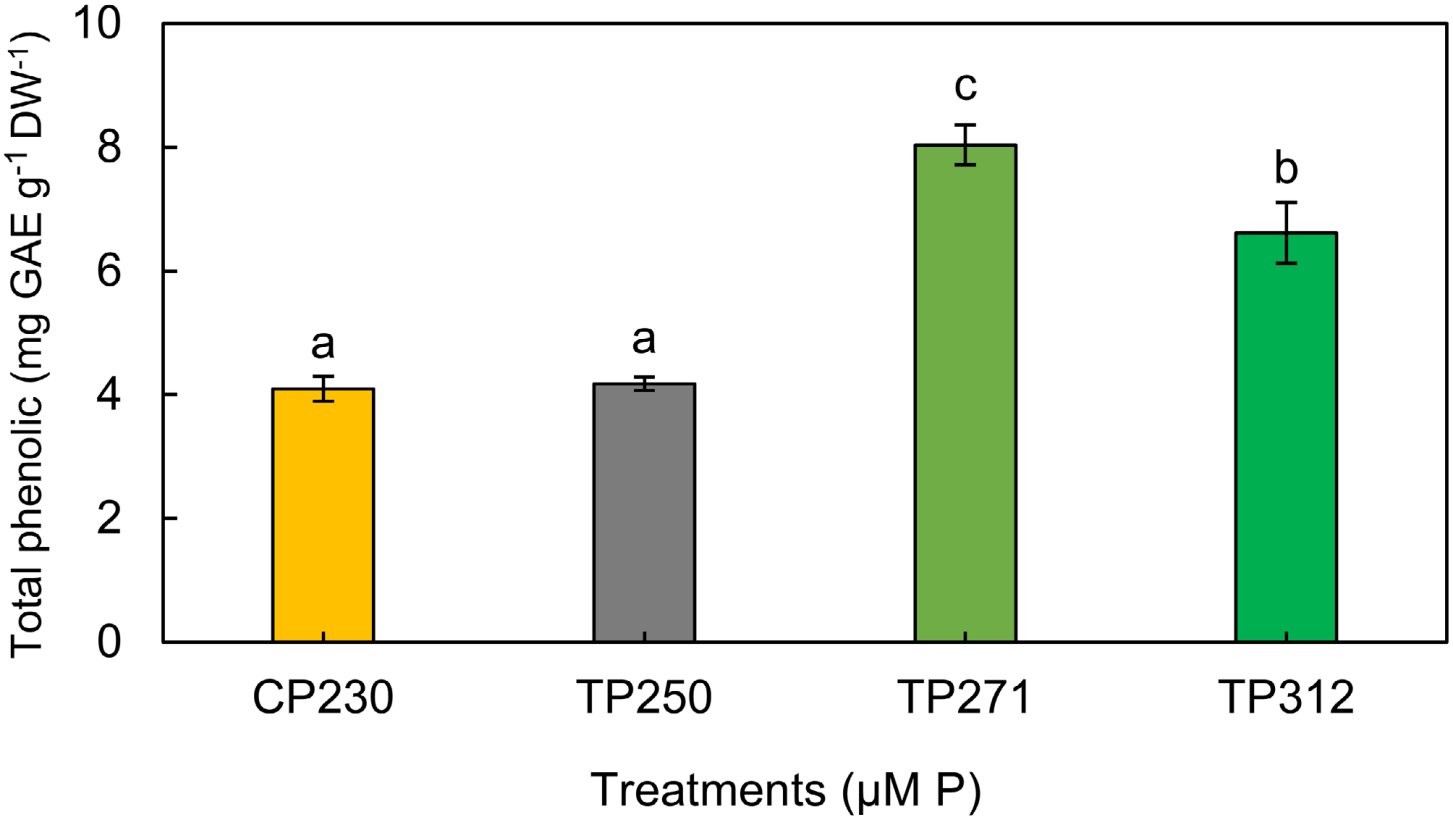
Effect of different concentrations of phosphorus on total phenolic content of *Anabaena* sp. Data represent means ± SD from three replicates. Bars with different letters indicate significant differences according to Duncan's test (*p* < 0.05).

Cyanobacteria produce phenolic compounds, which are significant biologically active substances. These compounds have garnered considerable attention because of their antioxidant activity and health benefits (Singh et al. 2017). Figure 3 illustrates the total phenolic content of the methanolic extract from *Anabaena* sp. biomass cultivated at varying phosphorus concentrations. The phenolic content exhibited substantial variation among different examined treatments (*p* < 0.05). The maximum total phenolic content was recorded as 8.04 ± 0.32 and 6.61 ± 0.49 mg GAE g^−1^ DW^−1^, corresponding to the TP_271_ and TP_312_ treatments, respectively. The lowest total phenolic content was achieved as 4.09 ± 0.20 and 4.17 ± 0.11 mg GAE g^−1^ DW^−1^ in the CP_230_ and TP_250_ treatments, respectively. Hamouda and Abou‐El‐Souod (2018) investigated the effects of different phosphorus concentrations (1, 2, 5, 7, and 10 mg L^−1^) on the growth and phenolic content of *Scenedesmus obliquus* . In their study, the maximum phenolic content was recorded at 7 mg P L^−1^, which also correlated with the maximum growth rate of *S. obliquus* at this concentration. The findings of the current study align with those of Goiris et al. (2015) concerning the impact of nutrient stress on the phenolic contents of microalgal species including *Phaeodactylum tricornutum* , *Tetraselmis suecica*, and *Chlorella vulgaris* . Their results showed that the phenolic contents of microalgal extracts dramatically reduced in nutrient‐deficient cultures. They also indicated that elevating nutrient levels in the culture medium to a certain threshold can enhance microalgal growth and biocompound accumulation, which varies depending on the species of algae.

#### 3.2.3 Identified Compounds of *Anabaena* sp. by GC–MS Analysis

**FIGURE 4:**
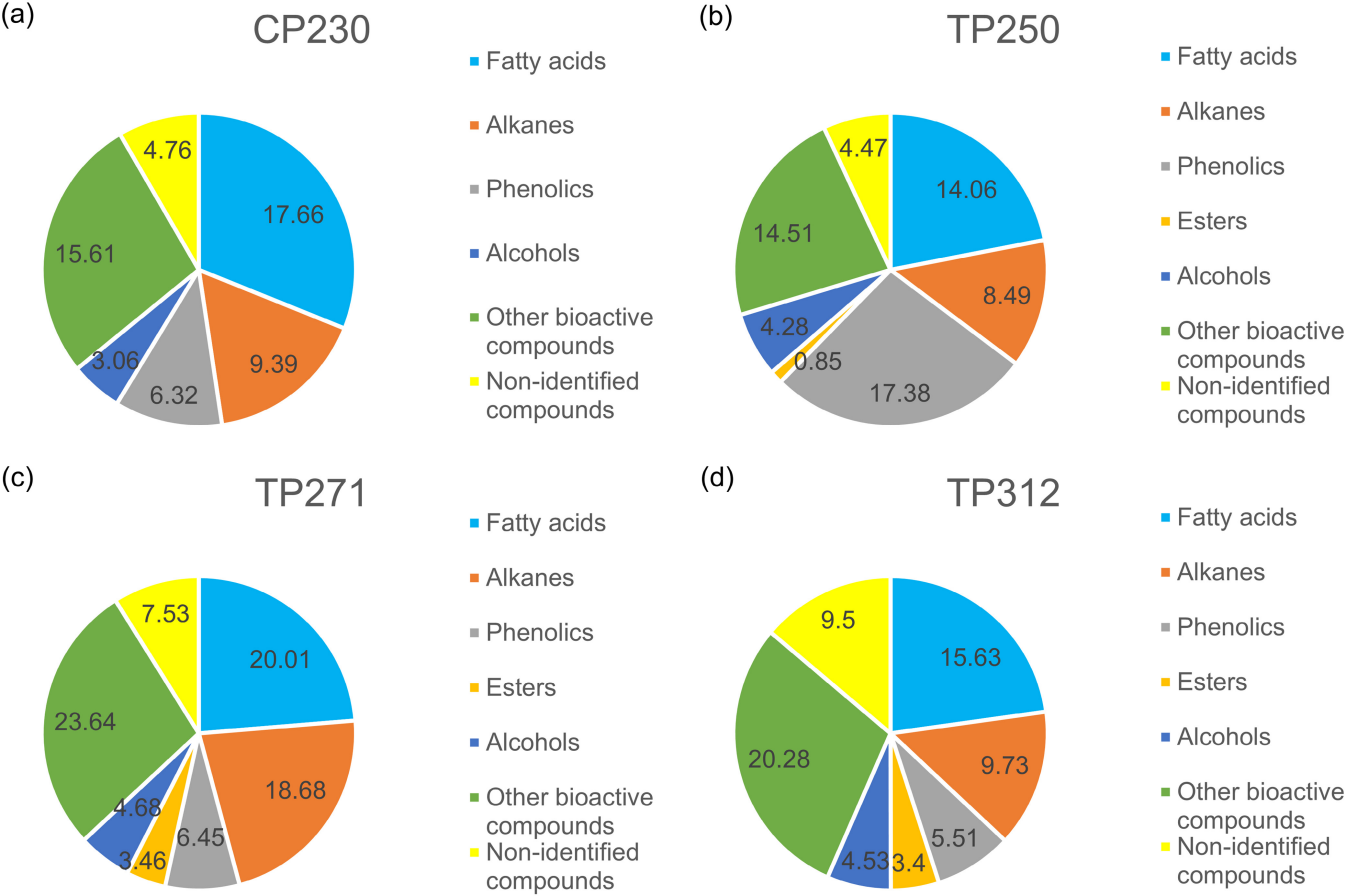
Amounts of major components identified in methanolic extract of *Anabaena* sp. in different experimental treatments (a‐d). The category named ‘Others’ includes: Gibberellic acid, propanoate, neophytadiene, N‐ethyl‐1,3‐dithioisoindoline, 1,3‐Bis(trimethyl)benzene, and 3‐Nitrophthalic acid.

Cyanobacteria produce various types of bioactive compounds, including phenols, alkaloids, saponins, tannins, terpenoids, flavonoids, and polysaccharides (Devi et al. 2020), which enable them to adapt to biological stress and environmental challenges, ultimately providing advantages for their survival (Seddek et al. 2019). Major phytochemical components present in the methanol extract of *Anabaena* sp., along with their molecular formulas and values in different examined treatments, are shown in Table 3. The GC–MS chromatogram of the methanol extract of *Anabaena* sp. showed the presence of several active principal compounds. The prevailing compounds were fatty acids, alkanes, alcohols, phenolics, and esters (Figure 4a–d), which could be responsible for the recorded biological activity of this species. The fatty acids of *Anabaena* sp. as bioactive compounds were 17.66%, 14.06%, 20.01%, and 15.63% at CP_230_, TP_250_, TP_271_, and TP_312_ treatments, respectively. Fatty acids may act as antimicrobial agents by disrupting the cell membrane of microorganisms, leaking or reducing nutrient absorption, and inhibiting cellular respiration (Saly and Gehan 2020). Kumar et al. (2011) stated that it is likely that fatty acids, by spreading in the peptidoglycan network of the microbial cell wall, cause disintegration and decomposition of the cell membrane. Alkanes were another group of identified compounds, with total amounts of 9.39%, 8.49%, 18.68%, and 9.73% at CP_230_, TP_250_, TP_271_, and TP_312_ treatments, respectively. Some alkanes, such as Eicosane, have been reported to have antifungal, antibacterial, antitumor, and cytotoxic properties (Saly and Gehan 2020; Octarya et al. 2021).

**TABLE 3:** Major components identified in *Anabaena* sp. extract based on percentage.

Major compounds	Treatments (μM P)			
	CP_230_	TP_250_	TP_271_	TP_312_
Hexadecanoic acid Methyl ester (C_17_H_34_O_2_)	10.89	9.55	12.93	11.13
Octadecenoic acid, methyl ester (C_19_H_34_O_2_)	3.98	1.38	3.65	2.30
9‐octadecenoic acid‐, methyl ester (C_19_H_36_O_2_)	2.79	3.13	3.43	2.20
*Σ* (Fatty acids methyl ester)	17.66	14.06	20.01	15.63
Dodecane, 2‐methyl‐6‐propyl (C_16_H_34_)	6.43	3.40	10.90	5.36
Eicosane (C_20_H_42_)	—	—	—	2.05
Heptadecane (CH_3_(CH_2_)_15_CH_3_)	—	2.31	3.03	
Hexadecane (C_16_H_34_)	2.96	2.78	4.75	2.32
*Σ* (Alkanes)	9.39	8.49	18.68	9.73
2‐Methyl‐1‐propanol (C_4_H_10_O)(Isobutanol)	1.37	0.98	2.26	1.19
1‐Pentanol (C_5_H_12_O)	1.69	0.81	2.42	1.70
3,7,11,15‐Tetramethyl‐2‐hexadecen‐1‐ol (C_20_H_40_O)	—	2.49	—	1.64
*Σ* (Alcohols)	3.06	4.28	4.68	4.53
Phenol, 2,4‐bis (1,1‐dimethyl ethyl) (C_14_H_22_O)	3.04	12.27	3.39	2.45
Methyl 3‐(3,5‐di‐tert‐butyl‐4 hydroxyphenyl) propionate (C_18_H_28_O_3_)	3.28	5.11	3.06	3.06
*Σ* (Phenolics)	6.32	17.38	6.45	5.51
Phthalic acid, butyl tetradecyl ester (C_26_H_42_O_4_)	—	0.85	3.46	3.40
*Σ* (Esters)	0.00	0.85	3.46	3.40
Gibberellic acid (C_19_H_22_O_6_)	—	—	—	2.06
Propanoate (C_3_H_5_O_2_)	4.52	—	—	—
Neophytadiene (*C*_*20*_ *H*_*38*_ *)*	—	5.35	3.55	3.48
N‐ethyl‐1,3‐dithioisoindoline (C_10_H_13_NS_2_)	—	—	—	3.02
3‐Nitrophthalic acid (C_8_H_5_NO_6_)	3.35	7.09	7.84	6.06
1,3‐Bis(trimethyl)benzene (C_12_H_22_Si_2_)	7.74	2.07	12.25	7.72
*Σ* (Other bioactive compounds)	15.61	14.51	23.64	20.28
*Σ* (Non‐identified compounds)	4.76	4.47	7.53	9.50

Other groups identified in the GC–MS results of the methanolic extract of *Anabaena* sp. were alcohols with the total values of 3.06, 4.28, 4.68, and 4.53, and phenolics with 6.32, 17.38, 6.45, and 5.51 at CP_230_, TP_250_, TP_271_, and TP_312_ treatments, respectively. Phenolic components are one of the most abundant classes of phytochemicals in algae and have potential antioxidant and antimicrobial ability (Saly and Gehan 2020; Devi et al. 2021; Idih et al. 2021). Antimicrobial properties have also been reported for alcohol compounds such as 1‐propanol, 2‐Methyl‐1‐propanol (isobutanol), 1‐Pentanol, 1‐Butanol, 2‐methyl‐ (S)‐, and 1‐Butanol, 3‐methyl by Saly and Gehan (2020). Other compounds identified in GC–MS analysis, such as Neophytadiene, have also been reported to have antioxidant and antibacterial properties (Sosa et al. 2016). The bioactive compounds differences at *Anabaena* sp. at various phosphorus treatments may be attributed to the conversion of these secondary metabolites based on the cellular requirements of cyanobacteria that help them during adaptation to the environment (Zyszka‐Haberecht et al. 2019).

#### 3.2.4 Antioxidant Activity

**FIGURE 5:**
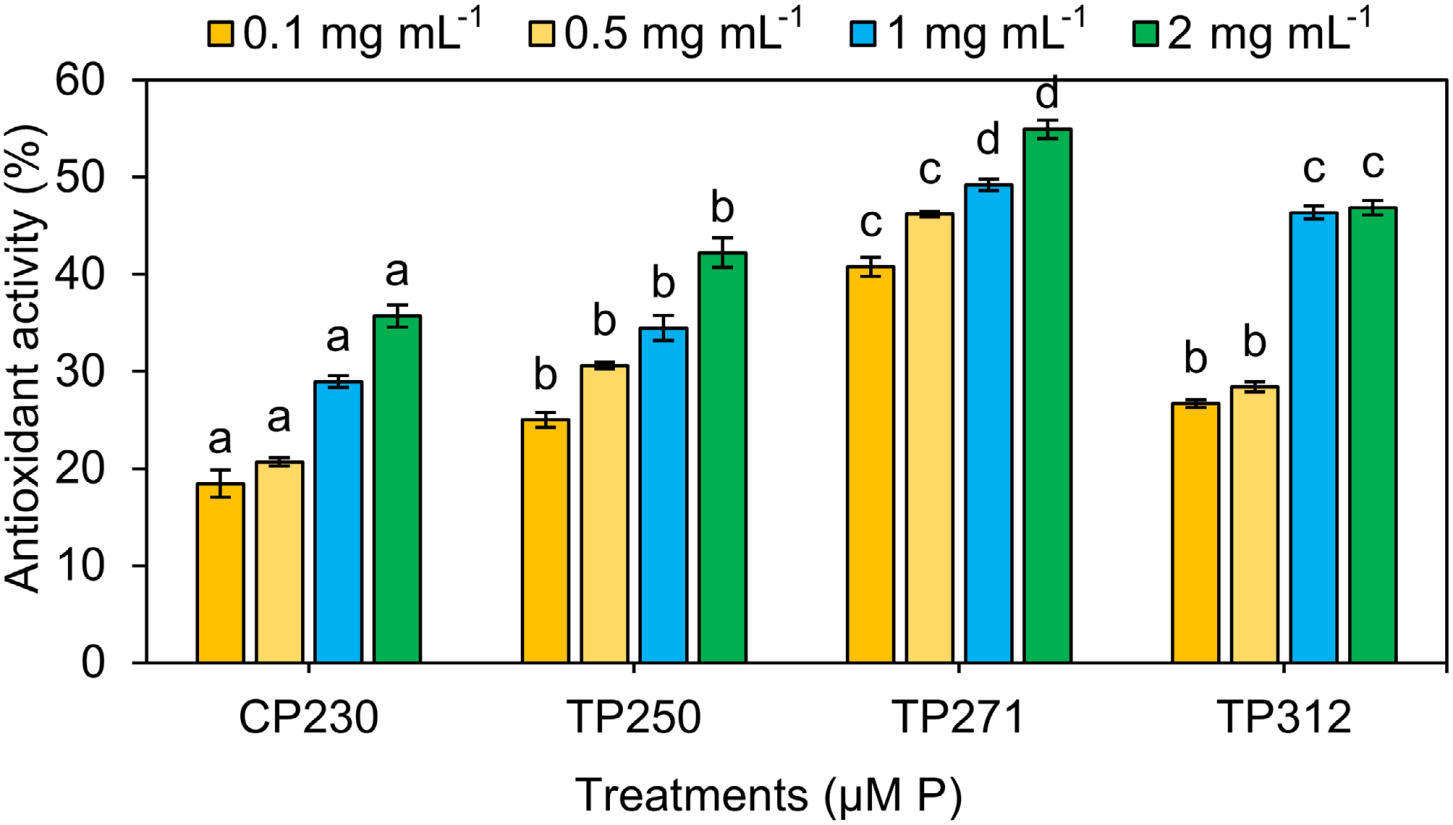
Effect of different methanolic extracts and media initial P concentrations on the antioxidant activity of *Anabaena* sp. Data represent means ± SD from three replicates. Bars with different letters indicate significant differences according to Duncan's test (*p* < 0.05).

Figure 5 illustrates the antioxidant activity of extracts from *Anabaena* sp. biomass, cultivated in media with varying phosphorus concentrations. The results of the DPPH assay indicated that the antioxidant efficacy of all treatments enhanced as the concentration of the extract increased from 0.1 to 2 mg mL^−1^. Consequently, the antioxidant performance in all treatments showed an increase with higher concentrations of the extract, with the peak antioxidant activity (54.94%) recorded in the TP_271_ treatment. These findings revealed that the antioxidant capacity of *Anabaena* sp. biomass extracts across various experimental treatments was influenced by both the concentration of the extract and the phosphorus levels in the treatments. According to Wickramasinghe et al. (2024), the presence of phenolic and flavonoid compounds in cyanobacterial extracts contributes to their antioxidant activity. Furthermore, Davodbasha et al. (Davoodbasha et al. 2018) stated that cyanobacterial compounds exhibit their antioxidant properties by donating hydrogen to free radicals, thereby converting them into non‐reactive species. Goiris et al. (2015) examined the impact of nutrient stress on the production of antioxidants in various microalgae species. They found that the overall efficiency of antioxidants is significantly greater under nutrient‐sufficient conditions compared to nutrient‐limited environments. Their findings indicated that the antioxidant efficacy in cultures with low and restricted phosphorus levels is 2–6 times lower compared to conditions with adequate phosphorus availability. Apel and Hirt (2004) noted that nutritional constraint in microalgae disrupts electron transfer from the photosystem to the electron transport chain. Consequently, this disruption leads to the formation of reactive oxygen species. A reduction in total antioxidant levels under low or nutrient‐deficient conditions may suggest that algae cells are more vulnerable to reactive oxygen species (Apel and Hirt 2004).

The methanolic extract of the TP_271_ treatment exhibited suitable IC50 values of 2.985 ± 0.02 mg mL^−1^, showing a significant difference compared to the other examined treatments (*p* < 0.05, Table 4). The IC50 values of DPPH for CP_230_, TP_250_, and TP_312_ treatments were determined to be 6.50 ± 0.01, 5.54 ± 0.09, and 4.14 ± 0.05 mg mL^−1^, respectively. The control treatment exhibited the lowest IC50, determined to be 2.18 times lower than the highest IC50 and 63 times lower than that of vitamin C (as a standard antioxidant) at 0.103 mg mL^−1^. Overall, elevated phosphorus levels (specifically TP_271_ and TP_312_) demonstrated enhanced antioxidant activity and IC50 compared to the control treatment and lower phosphorus levels. The findings of Hamouda and Abou‐El‐Souod (2018) demonstrated that the antioxidant activity of *S. obliquus* is influenced by its growth rate. In their study, the highest antioxidant activity and the highest growth rate were achieved with 7 mg L^−1^ phosphate concentration.

**TABLE 4:** IC_50_ of DPPH (mg mL^−1^) of methanolic extract *Anabaena* sp. grown in different phosphorus concentrations.

Treatments (μM P)	IC_50_ values (mg mL^−1^)
	Radical Scavenging Activity
TP_230_	6.50 ± 0.01^c^
TP_250_	5.54 ± 0.09^bc^
TP_271_	2.98 ± 0.02^a^
TP_312_	4.14 ± 0.05^b^
Vitamin C	0.103

*Note:* The values with the different letters are significantly different (*p* < 0.50).

#### 3.2.5 Antibacterial Properties

Cyanobacteria are prospective sources of novel bioactive compounds and have been extensively studied for their antibacterial properties (Marrez et al. 2017; Singh et al. 2017). Table 5 summarizes the findings of the antibacterial efficacy of methanolic extract of *Anabaena* sp. cultivated at varying phosphorus concentrations against *Staphylococcus aureus* and *Escherichia coli* . The results demonstrated that cyanobacterial extracts indicated varying levels of antibacterial activity, and the intensity of their inhibitory effect was contingent upon the concentration of phosphorus and the species of tested bacteria. *E. coli* exhibited greater sensitivity to the methanolic extract of *Anabaena* sp., with inhibition diameters ranging from 4 ± 0.2 to 9.3 ± 1.2 mm. It was reported by Chauhan et al. (2014) that the cyanobacterial extract of *Anabaena* sp. exhibited the highest antibacterial activity against *E. coli* compared to *E. aerogenes* . The findings indicated that TP_271_ and TP_312_ treatments had the highest antibacterial efficacy. Their inhibitory activity against *E. coli* was 9.30 ± 1.20 mm and 8.50 ± 0.50 mm, whereas against *S. aureus* it was 4.50 ± 0.90 mm and 4.50 ± 0.50 mm, respectively. No inhibition zone was detected for the proliferation of *S. aureus* in the control treatment. Therefore, it can be concluded that phosphorus positively influenced the inhibitory activity of the extracts against the examined bacteria. Hamouda and Abou‐El‐Souod (2018) investigated the influence of varying phosphorus concentrations on the antibacterial characteristics of *Scenedesmus obliquus* . Their findings showed that the antibacterial properties of *S. obliquus* enhanced as phosphorus concentration increased. The findings of the current study revealed that *S. aureus* exhibited reduced sensitivity to the methanolic extract of *Anabaena* sp. based on the antibacterial activity of various treatments examined. According to Duffy and Power (2001), a potential reason for the varying sensitivity between Gram‐positive and Gram‐negative bacteria to extracts may be attributed to the substantial differences in the outer layers of these bacteria.

**TABLE 5:** Antimicrobial activity and minimum inhibitory concentration of *Anabaena* sp. extract against *Staphylococcus aureus* and *Escherichia coli* (inhibition zone measured in mm).

Treatments (μM P)	Diameter of inhibition zone (mm)	Minimum Inhibitory Concentration (mg mL^−1^)		
	*Escherichia coli*	*Staphylococcus aureus*	*Escherichia coli*	*Staphylococcus aureus*
TP_230_	5 ± 0.50^a^	ND	1.25 ± 0.01^b^	5 ± 0.30^c^
TP_250_	4 ± 0.20^a^	2 ± 0.20^a^	1.25 ± 0.05^b^	2.5 ± 0.10^b^
TP_271_	9.30 ± 1.20^b^	4.50 ± 0.90^b^	0.625 ± 0.09^a^	1.25 ± 0.09^a^
TP_312_	8.50 ± 0.50^b^	4.50 ± 0.50^b^	0.625 ± 0.02^a^	1.25 ± 0.^05a^

*Note:* The values (mean ± SD) with the different letters are significantly different (*p* < 0.05).

Abbreviation: ND, not detected.

The methanolic extract of *Anabaena* sp. was tested for its minimum inhibitory concentration (MIC) on *E. coli* and *S. aureus* at serial concentrations ranging from 10 to 0.625 mg mL^−1^. The MIC point was determined to be the highest inhibitory dilution (Table 5). The TP_271_ and TP_312_ treatments showed superior antibacterial activity compared to other treatments tested against both bacterial strains. The MIC values of TP_271_ and TP_312_ treatments were 0.625 ± 0.09 and 0.625 ± 0.02 mg mL^−1^ for *E. coli* and 1.25 ± 0.09 and 1.25 ± 0.05 mg mL^−1^ for *S. aureus* , respectively. The MIC results demonstrated that elevating the phosphorus concentration positively influenced the antibacterial activity of the cyanobacterial extract from *Anabaena* sp. As phosphorus concentrations increased, the MIC was reduced, and the extracts exhibited enhanced inhibitory activity. The antibacterial activity of cyanobacterial extract is mostly due to the presence of several biochemical compounds exhibiting antibacterial properties, including saturated and polyunsaturated fatty acids (Alsenani et al. 2020; Frazzini et al. 2022).

### 3.3 The Effect of Phosphorus Concentrations on Toxin Compounds of Cyanobacterial Extracts

The microcystins (MCs) are considered to be the most hazardous groups of cyanotoxins produced by toxic cyanobacteria (Pham and Utsumi 2018). Production of MC is influenced by several environmental factors, including pH, light, nutrients, temperature, and dissolved oxygen (Li et al. 2017). Several studies have indicated that phosphorus plays a crucial role in the production of MC. Given that certain cyanobacteria possess the potential to fix nitrogen, it is commonly argued that phosphorus availability is a significant limiting factor for cyanobacterial proliferation and microcystin production (Li et al. 2023). The *Anabaena* sp. cultivated under varying phosphorus concentrations exhibited MC‐LR levels between 33.41 ± 0.97 and 300.50 ± 8.69 pg. mL^−1^ (Table 6). Table 6 also includes an acute toxicity test based on LC50‐96 h with lyophilized *Anabaena* sp. samples. The minimum LC50 value (indicating maximum toxicity) for 96 h was recorded for the aquatic extracts of *Anabaena* sp. obtained from TP_271_ treatments, equivalent to 271.12 ± 7.84 mg DW L^−1^. The quantity of MCs generated in the environment is influenced by the abundance of toxic cyanobacterial strains and the quantity of toxin produced by each cell (Chorus et al. 2001; Halstvedt et al. 2007). Conversely, Davis et al. (2009) observed that the algal growth rate and MCs content in a culture treated with phosphorus were notably elevated. Li et al. (2023) reported that phosphorus is an essential element in ATP synthesis. Phosphorus regulates the synthesis of MCs primarily by influencing ATP generation and the enzymatic activity involved in the MCs production pathway. Available reports indicated that a notable accumulation of cyanotoxins occurs in zooplankton tissue, potentially serving as a route for exposure to other invertebrates, fish, and even mammals (Ferrao‐Filho and Kozlowsky‐Suzuki 2011). This is due to the fact that zooplanktons consume cyanobacteria, store cyanotoxins, and transfer them throughout the food chain.

**TABLE 6:** MC‐LR of *Anabaena* sp. extracts in different phosphorus concentrations and median lethal concentrations (LC_50_) in 96 h exposure for the cladoceran, *Daphnia magna* .

Treatments (μM P)	MC‐LR (pg mL^−1^)	LC_50_‐96 h (mg DW L^−1^)
TP_230_	33.41 ± 0.97^a^	1790.31 ± 51.76^d^
TP_250_	68.41 ± 1.98^b^	1435.42 ± 41.50^c^
TP_271_	300.50 ± 8.69^d^	271.12 ± 7.84^a^
TP_312_	186.75 ± 5.40^e^	314.81 ± 9.10^b^

*Note:* Data represent mean ± SE. Different letters indicate significant differences between treatments (*p* < 0.05).

**FIGURE 6:**
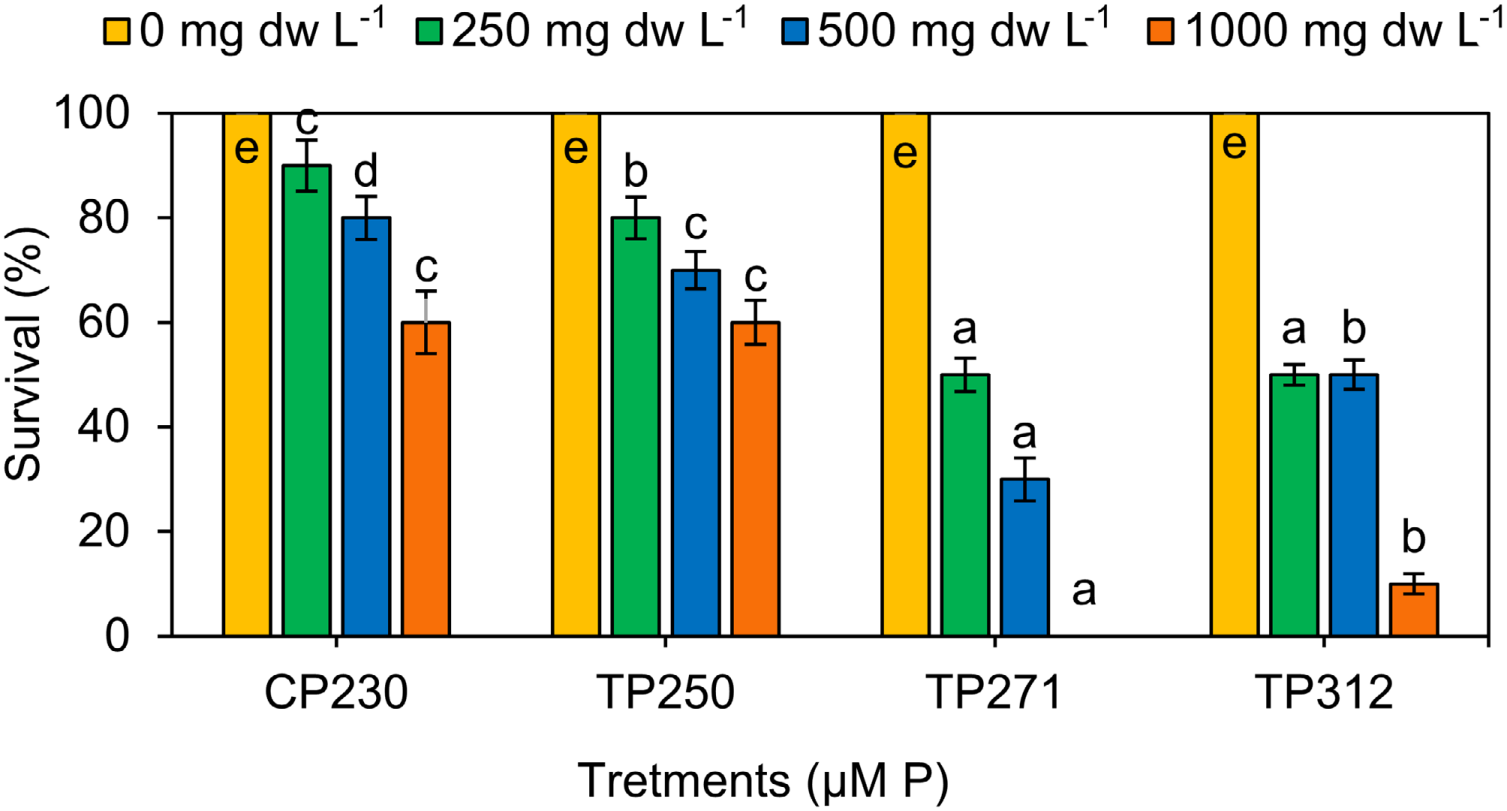
Effect of lyophilized biomass of *Anabaena* sp. grown in different phosphorus concentrations on survival of *D. magna* during 96 h of exposure. Data present means ± SD from three replicates. Bars with different letters indicate significant differences according to Duncan's test (*p* < 0.05).

Figure 6 illustrates the survival rate of *Daphnia magna* across various experimental treatments during a 96‐h period. The CP_230_ (control treatment) yielded the highest survival rate in *D. magna* of 60%, 80%, and 90% at aquatic extracts derived from 1000, 500, and 250 mg L^−1^ of dry cyanobacterial biomass, respectively (Figure 6). In general, the toxicity of *Anabaena* sp. increased as the concentration of the lyophilized biomass increased.

The findings indicated how phosphorus influences the growth of *Anabaena* sp., leading to an increase in toxin production. The control treatment exhibited the highest LC50 (indicating the lowest toxicity) at 1790.31 ± 51.76 mg DW L^−1^ (Table 6), which significantly differed from the other treatments. Therefore, it can be concluded that the toxicity level was increased with elevated concentrations of phosphorus and with prolonged exposure to *Anabaena* sp. biomass.

Herrera et al. (2015) examined the effects of phytoplankton extracts containing the toxin microcystin‐LR on the survival and reproduction of cladocerans. Their experiment demonstrated the extracts with the highest toxin concentration had the highest toxicities to cladocerans. Also, measurement of 48‐h LC50 showed consistent differences between cladoceran species. Species of zooplankton, the secondary metabolite profiles of cyanobacteria, and the method of exposure to extracted cyanotoxins can influence mortality rates (Pawlik‐Skowronska et al. 2019) and lead to reductions in reproduction rates and egg production in cladocerans (Herrera et al. 2015). For example, the acute toxicity of the aqueous extract of microcystin on freshwater cladocerans, including *Daphnia similis* , *Daphnia leavis*, and *Moina micrura* , at concentrations ranging from 25 to 1000 mg DW L^−1^ resulted in 100% mortality at 1000 mg DW L^−1^ for all three zooplankton species. The LC50‐48 h values were calculated to be 192.4, 270.5, and 413.9 mg DW L^−1^, respectively (Ferrao‐Filho et al. 2014).

## 4 Conclusions

This study demonstrated that phosphorus concentrations of cultivation medium (7.74, 8.38, and 9.66 mg P L^−1^) had a significant influence on the growth of *Anabaena* sp. as well as on bioactive compound production and toxin accumulation in the cyanobacterium biomass. Particularly, phosphorus supplementation at 8.38 mg L^−1^ maximized biomass production and improved the synthesis of valuable compounds, including total phenolic content, antioxidant capacity, and antibacterial efficacy against both Gram‐negative and Gram‐positive bacteria. However, phosphorus concentrations exceeding this threshold resulted in a decline in these beneficial properties. Notably, the phosphorus level that maximized growth and bioactivity also significantly elevated the toxicity of the extracts from *Anabaena* sp. biomass, as evidenced by heightened mortality in *Daphnia magna* assays. These findings highlighted the importance of carefully modulating phosphorus inputs to balance the biotechnological potential of *Anabaena* sp. with environmental safety considerations.

## Author Contributions

**Fatemeh Rostami:** writing – review and editing, writing – original draft, investigation, methodology, data curation. **Omidvar Farhadian:** writing – review and editing, validation, supervision, funding acquisition. **Nasrollah Mahboobi Soofiani:** review and editing, validation, supervision, methodology. **Mahmood Etebari:** review and methodology. **Amir Mahboubi Soufiani:** review and editing, validation.

## Ethics Statement

The authors have nothing to report.

## Conflicts of Interest

The authors declare no conflicts of interest.

## Data Availability

All raw data and materials used during the current study are available and can be shared on request.
